# Supplementary figures and images for: Mutations in the F protein of the live-attenuated respiratory syncytial virus vaccine candidate ΔNS2/Δ1313/I1314L increase the stability of infectivity and content of prefusion F protein
Source: PLoS One. 2024 Apr 9;19(4):e0301773. doi: 10.1371/journal.pone.0301773 (PMC11003679; doi:10.1371/journal.pone.0301773)

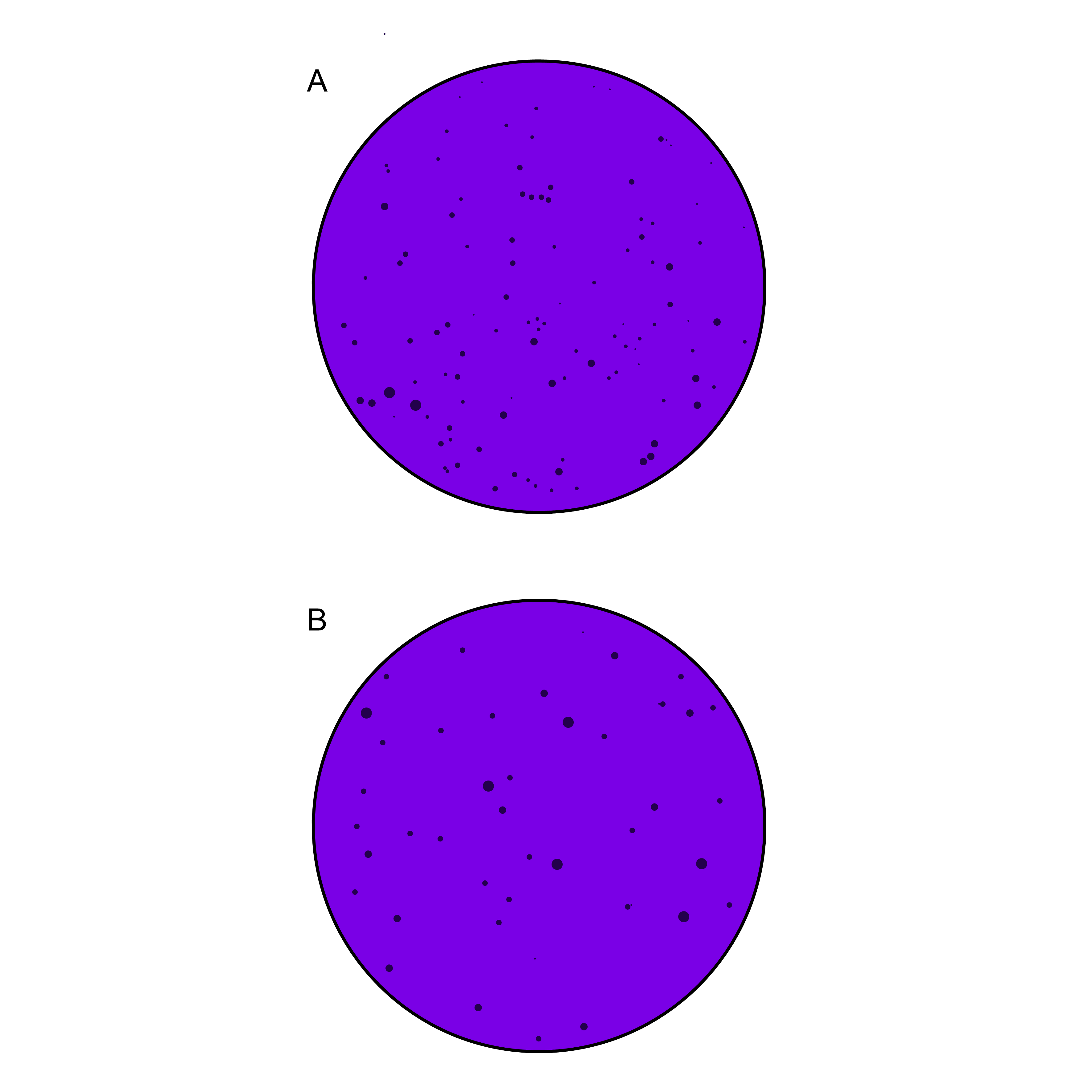

Supplement: S1 Fig — Images of representative wells from titrations of (A) original ΔNS2-L19F-4M material and (B) harvest from the 10th round of serial passage. Plaques were grown under overlay for 7 days at 32°C. (TIF) [file pone.0301773.s001.tif]
