# Supplementary material for: Mutations in the F protein of the live-attenuated respiratory syncytial virus vaccine candidate ΔNS2/Δ1313/I1314L increase the stability of infectivity and content of prefusion F protein
Source: PLoS One. 2024 Apr 9;19(4):e0301773. doi: 10.1371/journal.pone.0301773 (PMC11003679; doi:10.1371/journal.pone.0301773)
Supplement: S1 File — S1 Table. Data used to build Fig 2. S2 Table. Data used to build Fig 3A. S3 Table. Data used to build Fig 3B. S4 Table. Data used to build Fig 5A. S5 Table. Data used to build Fig 5B. S6 Table. Data used to build Fig 6A. S7 Table. Data used to build Fig 6B. S8 Table. Data used to build Fig 6C. (PDF) [file pone.0301773.s002.pdf]

Supplementary Information S1 File

Supplementary Table 1: Data used to build Figure 2

Day post infection	ΔNS2			ΔNS2-L19F-4M		
1	3.90E+05	4.15E+05	4.03E+05	1.28E+04	1.05E+04	1.10E+04
2	2.48E+06	2.15E+06	3.40E+06	1.00E+05	1.13E+05	1.58E+05
3	9.25E+06	1.05E+07	1.70E+07	5.45E+05	4.80E+05	4.90E+05
4	1.43E+07	1.65E+07	1.88E+07	1.80E+06	2.35E+06	2.58E+06
5	2.43E+07	2.28E+07	1.95E+07	4.23E+06	4.35E+06	3.95E+06
6	2.35E+07	2.48E+07	2.38E+07	5.43E+06	5.33E+06	5.53E+06
7	2.13E+07	1.90E+07	1.70E+07	8.10E+06	8.53E+06	2.13E+07
8	1.23E+07	1.13E+07		1.10E+07	8.25E+06	
9	1.20E+07	1.63E+07		9.75E+06	1.38E+07	
10	8.25E+06			1.18E+07	1.10E+07	

Supplementary Table 2: Data used to build Figure 3A

	A2			A2-L19F		
D25 / Palivizumab	1	1	1	1.34	1.27	1.14

Supplementary Table 3: Data used to build Figure 3B

	ΔNS2			ΔNS2-L19F-4M		
D25 / Palivizumab	1	1	1	5.6	4	4.4

Supplementary Table 4: Data used to build Figure 5A

Day post infection	A2		ΔNS2		ΔNS2-L19F-4M	
1	2.98E+04	2.40E+04	7.00E+03	1.63E+04	1.05E+03	1.98E+03
3	6.20E+06	6.40E+06	7.28E+04	4.50E+04	1.25E+04	3.08E+04
5	2.18E+06	2.55E+06	1.15E+04	1.33E+04	2.53E+04	3.05E+04
7	1.60E+06	1.15E+06	3.70E+03	1.80E+03	8.48E+03	5.58E+03
9	5.15E+05	7.03E+05	1.58E+03	1.53E+03	2.70E+03	1.60E+03

Supplementary Table 5: Data used to build Figure 5B

Day post infection	A2		ΔNS2		ΔNS2-L19F-4M	
1	6.28E+03	4.65E+04	9.25E+03	5.53E+03	4.25E+04	1.28E+04
3	5.55E+06	5.58E+06	2.35E+03	2.18E+03	9.25E+02	3.50E+02

5	1.60E+06	1.50E+06	1.15E+03	2.18E+03	6.25E+02	3.00E+02
7	8.25E+05	1.55E+06	1.18E+03	8.75E+02	3.50E+02	3.00E+02
9	3.05E+05	2.83E+05	6.25E+02	5.00E+02	1.15E+03	5.75E+02

Supplementary Table 6: Data used to build Figure 6A

Day post immunization	RSV rA2				ΔNS2				ΔNS2-L19F-4M			
1	5	5	5	5	5	5	5	5	5	5	5	5
2	5	5	45	15	10	5	5	5	5	5	5	10
3	1310	575	355	200	5	5	45	5	5	5	5	25
4	27000	6200	9850	8050	60	10	580	5	5	10	5	10
5	22500	115	8600	865	25	5	1600	25	20	1400	220	1000
6	15500	7500	4900	7850	130	110	9400	160	45	3300	210	740
7	3550	2700	4200	7650	20	1600	13000	5300	40	2500	1200	6600
8	12000	1500	485	230	150	1800	15000	7300	55	3000	880	85
9	2470	85	3580	45	860	2300	47000	9100	170	870	6300	15000
10	95	90	400	10	370	3400	11000	1600	320	3400	5300	15000
12					200	55	840	1400	210	720	85	230
14	5	5	5	5	5	5	5	5	25	5	5	5

Supplementary Table 7: Data used to build Figure 6B

Day post immunization	RSV rA2				ΔNS2				ΔNS2-L19F-4M			
2	1030	960	350	80	10	10	10	10	10	10	10	10
4	6100	1010	50	3500	10	10	10	10	10	10	10	10
6	19800	22200	710	49000	20	10	20	10	10	10	10	70
8	29200	2360	900	1850	10	10	80	10	10	10	10	50
10	420	620	720	10	70	10	20	400	10	10	40	1320
12					10	10	10	280	10	10	10	10
14	10	10	10	10	10	10	10	10	10	10	10	10

Supplementary Table 8: Data used to build Figure 6C

Day post immunization	RSV rA2				ΔNS2				ΔNS2-L19F-4M			
21	7.10	8.10	5.90	7.70	6.90	6.60	8.00	9.80	8.50	7.60	3.50	8.90
28	6.70	8.70	5.90	8.80	7.50	9.30	8.60	10.10	9.20	7.60	6.10	9.60
