# Supplementary material for: Mutations in the F protein of the live-attenuated respiratory syncytial virus vaccine candidate ΔNS2/Δ1313/I1314L increase the stability of infectivity and content of prefusion F protein
Source: PLoS One. 2024 Apr 9;19(4):e0301773. doi: 10.1371/journal.pone.0301773 (PMC11003679; doi:10.1371/journal.pone.0301773)
Supplement: Attachment — Submitted filename: Response to Reviewers.docx [file pone.0301773.s003.docx]

Sanofi

200 West St.

Waltham MA 02451

Feb 15, 2024

Dear Editors,

First, I would like to express our sincere gratitude to the reviewers for their invaluable time in evaluating our manuscript: PONE-D-24-02628 “Mutations in the F protein of the live-attenuated respiratory syncytial virus vaccine candidate ∆NS2/∆1313/I1314L increase the stability of infectivity and content of prefusion F protein”.

Regarding all comments received, we have thoroughly addressed each point and provided comprehensive responses, which can be found below. Furthermore, we have revised the manuscript to incorporate the additional requirements outlined in the decision letter from PLOS ONE. We are confident that the revised manuscript now meets all necessary criteria for publication in PLOS ONE.

Sincerely,

Dr. Linong Zhang

Senior expert scientist, Sanofi

**Responses to Reviewers' comments:**

- Line 192: please clarify why the cultures were transferred from 32C to 37C after 2 days – did that alter cell numbers appreciably?
- Answer:
  - As explained in lines 385 – 387 “In the second experiment, the HAE were infected and cultured at 32°C for 2 days, then transferred to 37°C to better simulate the conditions encountered in vivo as RSV spreads from the upper to the lower airways”.
  - The HAE cultures were fully differentiated before infection (line 380). Fully differentiated HAE cultures are 100% confluent and there is no further cell division during the infection time course. Therefore, transfer of the cultures from 32°C to 37°C does not alter cell numbers.
- Line 275: please indicate/comment/reference why ΔNS2-L19F-4M growth was delayed
- Answer:
  - The delay in viral growth could be due to the slower spread of infection by ΔNS2-L19F-4M, possibly influenced by a more stable form of the fusion protein responsible for mediating virus infection in Vero cells. However, despite this delay, ΔNS2-L19F-4M ultimately reached the same level of virus peak titers as the parental virus.
- Line 362: please comment on why both vaccine candidates' viral shedding peaks were substantially later (d 9,10) than those in the RSV A2 group. Does cell cytotoxicity correlate with this difference?
- Answer:
  - The delay in peak shedding for both vaccine candidates is primarily attributed to the lack of IFN antagonist effect mediated by the NS2 gene, and to the deletion of codon 1313 of the RSV polymerase protein L. The NS2 gene was intentionally deleted from the two attenuated strains. RSV with deletion of the NS2 gene grows more slowly and to lower titers in interferon competent cells, e.g., A549 or Hep-2 ([N Teng](https://pubmed.ncbi.nlm.nih.gov/?term=Teng+MN&cauthor_id=9847352), [P L Collins](https://pubmed.ncbi.nlm.nih.gov/?term=Collins+PL&cauthor_id=9847352). Altered growth characteristics of recombinant respiratory syncytial viruses which do not produce NS2 protein. J Virol. 1999 Jan;73(1):466-73). Both vaccine candidates are also attenuated by a deletion of codon 1313 of the polymerase L ORF, which is attenuating and confers temperature sensitivity to RSV. When grown at the fully permissive temperature of 32°C in Vero cells, which do not have the ability to produce Type I interferon, the replication kinetics of RSV/ΔNS2/Δ1313/I1314L are highly similar to those of RSV A2 (which is beneficial for vaccine manufacture). However, in animal models and clinical studies ([C K Cunningham](https://pubmed.ncbi.nlm.nih.gov/?term=Cunningham+CK&cauthor_id=35732186), [R A Karron](https://pubmed.ncbi.nlm.nih.gov/?term=Karron+RA&cauthor_id=35732186) Evaluation of Recombinant Live-Attenuated Respiratory Syncytial Virus (RSV) Vaccines RSV/ΔNS2/Δ1313/I1314L and RSV/276 in RSV-Seronegative Children. J Infect Dis. 2022 Dec 13;226(12):2069-2078), shedding of RSV/ΔNS2/Δ1313/I1314L peaks later than wildtype RSV A2 would. This is one of the key features of attenuation.
- Please speculate how the adventitious mutation K75N in the F protein affects plaque size/spread
- Answer:
  - We speculate that the K75N mutation may favor more efficient fusion activity, thereby triggering fusion events leading to faster viral spread and increased plaque size. However, further investigation is needed to confirm and elucidate the underlying mechanisms.

**Additional requirements:**

1) Please ensure that your manuscript meets PLOS ONE’s style requirements, including those for file naming. The PLOS ONE style templates can be found at <https://journals.plos.org/plosone/s/file?id=wjVg/PLOSOne_formatting_sample_main_body.pdf>

**We reformatted the manuscript to ensure alignment with PLOS ONE’s style requirements, including those for file naming.**

2) In order to comply with PLOS ONE's guidelines for non-human primate experiments ([http://journals.plos.org/plosone/s/submission-guidelines#loc-non-human-primates](https://protect-de.mimecast.com/s/gjb-Ck2Y4XcAxjqrGI8iJ9Q?domain=journals.plos.org)), please provide additional details regarding housing conditions, feeding regimens, environmental enrichment, and all relevant steps taken to alleviate suffering. Also indicate how often animal care staff monitored the health and well-being of the animals and the criteria used to make such assessments. Lastly, specify the disposition of animals at the end of the study (e.g. euthanasia, returned to home colony, etc.).

**We added additional details on NHP housing conditions, feeding regimens, environmental enrichment etc. to the Materials and methods lines 251 – 253 and the disposition of animals at the end of the study to lines 260 and 261.**

3) In your Methods section, please include a comment about the state of the animals following this research. Were they euthanized or housed for use in further research? If any animals were sacrificed by the authors, please include the method of euthanasia and describe any efforts that were undertaken to reduce animal suffering.

**Information on the disposition of animals at the end of the study was added to Materials and methods lines 260 and 261.**

4) We note that your Data Availability Statement is currently as follows: [All relevant data are within the manuscript and its Supporting Information files.] Please confirm at this time whether or not your submission contains all raw data required to replicate the results of your study. Authors must share the “minimal data set” for their submission. PLOS defines the minimal data set to consist of the data required to replicate all study findings reported in the article, as well as related metadata and methods ([https://journals.plos.org/plosone/s/data-availability#loc-minimal-data-set-definition](https://protect-de.mimecast.com/s/BewBClRZ4EsmQ8zA4IVy7v4?domain=journals.plos.org)).

For example, authors should submit the following data:

- The values behind the means, standard deviations and other measures reported;

- The values used to build graphs;

**We added a Supplementary Information file (S1 File) containing the values behind the means and standard deviations used to build graphs.**

5) Please include a separate caption for each figure in your manuscript.

**We inserted a separate caption for each figure into the body of the manuscript.**

6) Please include captions for your Supporting Information files at the end of your manuscript, and update any in-text citations to match accordingly. Please see our Supporting Information guidelines for more information: [http://journals.plos.org/plosone/s/supporting-information](https://protect-de.mimecast.com/s/rU6gCnR246sg826KBIvq2Eg?domain=journals.plos.org).

**We added captions for our Supporting Information files to the end of the manuscript as per the supporting information guidelines.**

7) Please review your reference list to ensure that it is complete and correct. If you have cited papers that have been retracted, please include the rationale for doing so in the manuscript text, or remove these references and replace them with relevant current references. Any changes to the reference list should be mentioned in the rebuttal letter that accompanies your revised manuscript. If you need to cite a retracted article, indicate the article’s retracted status in the References list and also include a citation and full reference for the retraction notice.

**We reviewed the reference list to ensure that it is complete and correct. We have not made any changes to the list, and it does not contain any retracted papers.**
