# Supplementary material for: Mutations in the F protein of the live-attenuated respiratory syncytial virus vaccine candidate ΔNS2/Δ1313/I1314L increase the stability of infectivity and content of prefusion F protein
Source: PLoS One. 2024 Apr 9;19(4):e0301773. doi: 10.1371/journal.pone.0301773 (PMC11003679; doi:10.1371/journal.pone.0301773)
Supplement: Attachment — Submitted filename: Response to Reviewers.docx [file pone.0301773.s004.docx]

Sanofi

200 West St.

Waltham MA 02451

March 18, 2024

Dear Editors,

I would like to extend our appreciation once more to the reviewers for their critical comments and feedback on our revised manuscript: PONE-D-24-02628 “Mutations in the F protein of the live-attenuated respiratory syncytial virus vaccine candidate ∆NS2/∆1313/I1314L increase the stability of infectivity and content of prefusion F protein”.

Regarding all new comments received, we have thoroughly addressed each point, provided comprehensive responses which can be found below and made the suggested revisions to the manuscript including incorporation of additional references. We are confident that the revised manuscript now meets all necessary criteria for publication in PLOS ONE.

Sincerely,

Dr. Linong Zhang

Senior expert scientist, Sanofi

**Responses to Reviewers' comments:**

Major Comments:

- The authors claim in their abstract that “these results suggest that ΔNS2-L19F-4M is an improved live attenuated vaccine candidate that merits further evaluation in a clinical trial in humans”. What the authors have shown is that even an increase in the expression of the prefusion/fusion protein ratio and increased virus stability in tissue culture does not result in any measurable differences in vivo. Thus, the conclusion should be that ΔNS2-L19F-4M does not promise to be superior to ΔNS2.
- Answer
  - In the Discussion section, we had previously explained how the increased stability of the ΔNS2-L19F-4M candidate could potentially improve the manufacturability, storage and distribution of the ΔNS2 vaccine currently in phase III clinical trials (lines 464 -476). In response to the reviewer’s comment, we have modified the abstract (Lines 47 and 48) and the concluding paragraph of the Discussion (Lines 521 – 524) to make this critical point more explicit.
- It is unfortunate that the authors did not challenge the immunized animals to compare immunization efficiency. If these data are available, they should be added to the manuscript.
- Answer
  - The AGM model is well suited to investigating differences in immunogenicity and attenuation of live attenuated RSV vaccine candidates delivered intranasally. However, in our previous AGM challenge studies, we observed no distinctions among different live attenuated RSV vaccine candidates regarding protection, as all candidates provided complete protection against both upper and lower airway virus shedding in AGMs. This outcome may be linked to the semi-permissive nature of RSV infection in AGMs and the strong mucosal immunity induced by the vaccine candidates through intranasal immunization. Consequently, we deemed it unnecessary to conduct AGM challenge studies aimed at distinguishing between the live attenuated vaccine candidates.

Minor comments:

- Line 75: update that a second vaccine is on the market.
- Answer
  - We re-wrote lines 74 – 80 to explicitly list all 3 products currently licensed to protect infants from RSV, adding a reference for Palivizumab (Ref #12).
- Line 75-76: vaccination is not a passive approach, rephrase
- Answer
  - We re-wrote lines 74 – 80 to clarify that only mAb prophylaxis is a passive approach.
- Line 100-101 provide reference
- Answer

We added references for G mediated attachment via CX3C on HAE (Ref #28-30).
